# Reliability of the Soleus H-Reflex in Different Sitting Postures

**DOI:** 10.3390/medsci8040048

**Published:** 2020-11-25

**Authors:** Hamad S. Al Amer, Mohamed A. Sabbahi, Sharon L. Olson

**Affiliations:** 1 Department of Physical Therapy, Faculty of Applied Medical Sciences, University of Tabuk, Tabuk 71491, Saudi Arabia; 2 School of Physical Therapy, Texas Woman’s University, 6700 Fannin Street, Houston, TX 77030, USA; msabbahi@twu.edu (M.A.S.); solson@twu.edu (S.L.O.)

**Keywords:** H-reflex, soleus, sitting, electromyography, reliability

## Abstract

The Soleus (SOL) Hoffmann reflex (H-reflex) is commonly recorded in sitting position. However, the reliability of recording is unknown. We assessed the reliability of SOL H-reflex amplitude measurements across multiple traces and sessions during erect, slumped, and slouched sitting postures using the generalizability theory. Five traces of the SOL H-reflex maximum amplitude (H_max_) were recorded from 10 healthy participants during erect, slumped, and slouched sitting postures in two sessions. A decision study analysis was then conducted to calculate the reliability coefficients of the H_max_ for five traces and two sessions and to mathematically calculate the coefficients for seven and ten traces, and one and three sessions in the three sitting postures. For five traces and two sessions, the results showed reliability coefficients between 0.970 and 0.971, 0.980 and 0.979, and equal to 0.943 for erect, slumped, and slouched sitting, respectively. Averaging five traces of the H_max_ in a single recording session was sufficient to obtain acceptable reliability in the three sitting postures (reliability range, 0.892–0.988). It was concluded that the SOL H_max_ can be recorded during erect, slumped, and slouched sitting postures with adequate reliability.

## 1. Introduction

The Hoffmann reflex (H-reflex) is a compound muscle action potential that can be recorded from several skeletal muscles [1]. The H-reflex is elicited by sending an electrical stimulus along the large fast-conducting Ia sensory fibers to the alpha motoneurons (α-MNs) in the ventral horn of the spinal cord. The activation of α-MNs in the spinal cord generates an action potential traveling via α-motor axons toward the neuromuscular junction and produces the reflex contraction of the muscle, as well as the twitch response shown in the electromyography (EMG) unit (i.e., the H-reflex) [2]. The H-reflex onset latency and amplitude are the commonly used parameters in H-reflex studies. The H-reflex latency is the time between the initial deflection of the stimulus artifact and the initial deflection of the action potential of the H-reflex. The H-reflex amplitude represents the excitability of α-MN pool of the tested muscle [1,2]. The H-reflex recording is a valuable method to assess the integrity of conductivity through the reflex pathway and α-MN pool excitability [1,2].

Several factors can modulate the H-reflex amplitude and therefore, may affect the reliability of recordings. These factors may include, but are not limited to, joint angle [3,4,5], muscle activity [3,4,6], and head position and vestibular inputs [7,8,9]. Other sources of the H-reflex amplitude modulation are the postural and loading changes of the spine. The H-reflex amplitude is sensitive to changes in the magnitude of the mechanical load on the vertebral column and/or spinal nerve roots [7,10,11]. It is also modulated by positional changes and postural modifications of the spine [12].

Recording the H-reflex parameters from patients and healthy individuals in a sitting position is a common clinical procedure, e.g., [5,13,14]. In fact, the sitting position was identified as an ideal posture to record the soleus (SOL) H-reflex parameters [1]. This will be more convenient for the elderly as well as patients with respiratory problems and some neurological conditions such as Parkinson’s disease [15]. At the same time, different sitting postures have been shown to exhibit variability in the spinal alignment [16,17] and the magnitude of spinal loading [18,19]. However, data on the effects of trunk posture on the reliability of SOL H-reflex amplitude recordings in sitting position are lacking. To our knowledge, no published study has examined the stability and variability of the SOL H-reflex amplitude recordings during various sitting postures. In this study, we aimed to assess the reliability of the SOL H-reflex amplitude measurements across sessions and measurement traces during erect, slumped, and slouched sitting positions using the generalizability theory.

## 2. Materials and Methods

### 2.1. Participants

Ten healthy adult males were recruited for this study using convenience sampling. The anthropometric data of the participants are presented in Table 1. Participants were included based on the following criteria: no complaint of low back pain with or without leg pain; no injury to the ankle and/or foot at the time of the study; and no history of back or leg surgeries, peripheral neuropathy, upper motor neuron lesion, systemic or metabolic diseases (e.g., rheumatoid arthritis, diabetes mellitus), or cancer. Participants’ approvals for participation were obtained using written informed consent prior to the procedures. This study was approved by the Institutional Review Board of Texas Woman’s University, Houston center.

### 2.2. SOL H-Reflex Stimulation and Recording

**Figure 1:**
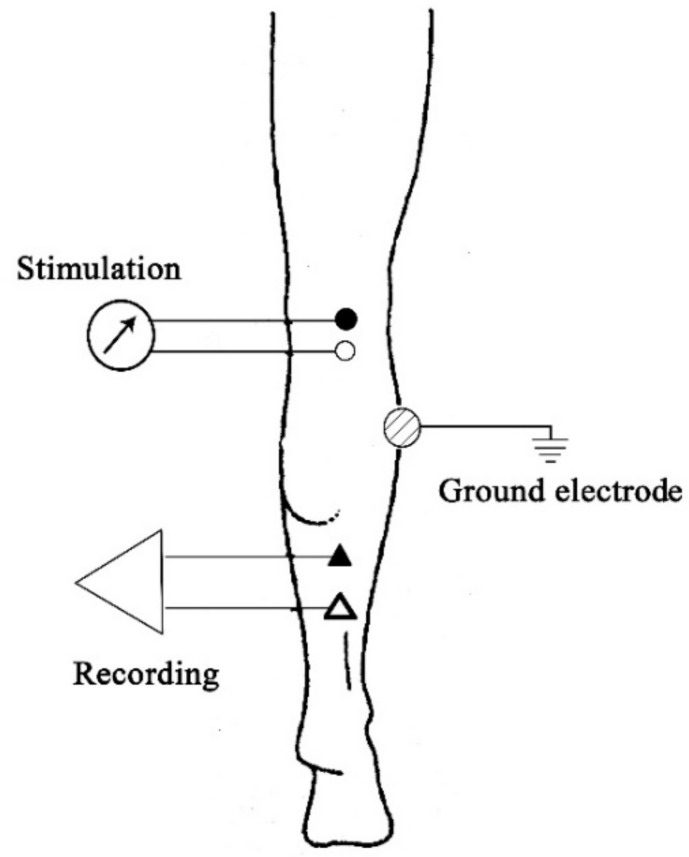
Location of the stimulating and recording electrodes of the soleus H-reflex. Black circle and triangle indicate the active electrodes of the stimulating and recording electrodes, respectively.

A Cadwell Sierra II EMG unit (Cadwell Laboratories, Inc., Kennewick, WA, USA), including a Sierra II 4-channel amplifier, was used to record the SOL H-reflex amplitude. Two silver–silver chloride (Ag–AgCl) bar electrodes were used for stimulation and recording. A 2 cm-diameter metal electrode was used as a ground electrode. The EMG unit setup was as follows: sensitivity/gain, 1–5 mV/division; and filter setting, 10 Hz–10 kHz [20]. The electrodes were placed while the participant was in prone position. Prior to the electrode placement, the skin areas where the electrodes were to be placed were gently rubbed with fine sand paper and cleaned with an alcohol pad to reduce skin impedance. The electrodes were then attached to the skin using 3-M hypoallergenic tape according to the following description. To stimulate the tibial nerve, the stimulating bar electrode was placed longitudinally at the midline of the popliteal fossa with the cathode (active) proximal to the anode (reference). The stimulating electrode delivered the percutaneous electrical stimuli of 1 ms square-wave pulses. The stimulation intensity was selected to produce maximal H-reflex amplitude (H_max_) together with minimal M-wave as seen in the earlier phase of the recruitment curve. The recording bar electrode was attached longitudinally 2 cm distal to the bifurcation of the gastrocnemius muscle in the midline and in line with the Achilles tendon. The recording electrode was attached so that the reference electrode was distal to the active electrode. The ground electrode was affixed to the lateral aspect of the leg midway between the stimulating and recording electrodes to reduce background noise [20] (Figure 1). Conductive gel was used to ensure that all electrodes achieve maximum conductivity. Participants were instructed to maintain the same arm and leg positions as much as possible throughout the test. To prevent vestibular influence on the SOL H-reflex amplitude, measurements were taken 3 min after the participants assumed the required position to allow for vestibular stability. Participants were also instructed to look forward at the eye level and maintain constant, midway head position [9]. To prevent the post-activation depression of reflex amplitude, an interstimulus interval of 10 s was employed [21].

### 2.3. Experimental Procedure

**Figure 2:**
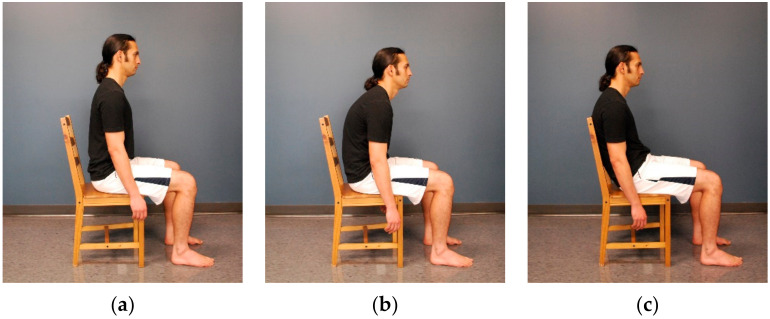
Testing sitting postures: (**a**) erect sitting; (**b**) slumped sitting; and (**c**) slouched sitting.

The SOL H-reflex amplitude was recorded from each participant’s right leg while sitting on a 45 cm high wood chair without armrests. The chair’s backrest was fixed at an inclination of 100° and was 47 cm high from the seat. No seat or backrest cushions were attached to the chair during testing. The SOL H-reflex was recorded in erect, slumped, and slouched sitting postures. In erect sitting, the participant was instructed to actively straighten the back and shoulders as much as possible without using the backrest, with the arms hanging at the sides (Figure 2a). In slumped sitting, the participant was instructed to forward slump the back and shoulders as much as possible, with the arms hanging at the sides (Figure 2b). In slouched sitting, the participant was instructed to slouch into the chair by leaning the trunk backward against the backrest until the position that provided maximal comfort for the participant was reached, with the arms hanging at the sides (Figure 2c). In all sitting postures, the participant was instructed to sit barefoot, maintain both feet flat on the floor and thighs at hip width, and distribute the weight evenly on both the lower limbs. When necessary, the height of the chair’s seat was adjusted, and the feet were supported using 2 inch wood steps to achieve ankle and knee joint angles at 90° in all sitting postures and hip at 90° in erect and slumped postures. A goniometer was used to ensure that the right angle of each joint was reached. To control for possible order effects, the administration of testing conditions was randomized. Two sessions of the SOL H-reflex recording in each sitting position were conducted. After the first session, the electrodes were removed, skin was cleaned, and the participants were given a 5 min rest period. The entire procedure was then repeated as the second session. The electrode sites were marked using a pen to ensure the same electrode location during the two sessions.

### 2.4. Statistical Analysis

Five traces of the SOL H_max_ were recorded in each session. Seven sources of the variability of the recordings were identified and analyzed for each sitting posture. These sources of variability included persons, sessions, traces, persons by sessions, persons by traces, sessions by traces, and the three-way interaction among person, session, and traces in addition to the residual. A brief explanation of each source is stated in Table 2. The estimated variance components for each source of variability were calculated using a two-facet, crossed, mixed generalizability (G)-study. Negative estimates of variability due to sampling error were set to zero in order to complete the analysis [22,23].

Based on the information obtained from the G-study, a decision (D)-study was then performed to calculate the G and Phi (Φ) coefficients of the SOL H_max_ recordings in the three sitting postures. The standard errors of measurement (SEM) and 95% confidence intervals (CI) of the G and Φ coefficients were also calculated. Data were analyzed using GENOVA (version 3.1; Center for Advanced Studies in Measurement and Assessment, Iowa City, IA, USA).

G-theory was used instead of the classical test theory for two major reasons. First, G-theory analyzes and provides estimates for each source of variability separately. This would provide the opportunity to determine which source has contributed the most to measurement error. Second, G-theory mathematically calculates the reliability coefficients when adding or reducing trials/items without the need to actually perform those alterations in practice. In this study, we measured the reliability for five traces and two sessions and mathematically calculated the coefficients for seven and ten traces and one and three sessions.

G and Φ coefficients range from 0 to 1 and are analogues to the reliability coefficient in classical test theory. The G coefficient reflects the reliability of measurement used for the relative decision, in which the aim of that measurement is to compare the score of one individual to the others. On the other hand, Φ coefficient reflects the reliability of measurement used for absolute decision, in which the aim of that measurement is to compare the score of one individual to a standard, regardless of other individuals’ scores [23]. G and Φ values are equivalent to the reliability coefficients in classical test theory [23]. Therefore, these values were interpreted as follows: <0.50, poor; 0.50–0.75, moderate; 0.75–0.90, good; and >0.90, excellent [24].

## 3. Results

The means and standard deviations of the SOL H_max_ for traces and sessions in each sitting posture are shown in Table 3. The estimated variance components and the percentage of variability for each source of variability in the three sitting postures are presented in Table 4. The G and Φ coefficients, SEM, and 95% CI for the SOL H_max_ recordings in the three sitting postures with various levels of sessions (1, 2, and 3) and measurement traces (5, 7, and 10) are presented in Table S1.

### 3.1. Results of the G-Study

As shown in Table 4, the results of the G-study showed that the highest portion of the estimated variance in the three sitting postures was due to people’s variability (i.e., true score variance). This was followed by person-by-session interaction variance across the three sitting postures, accounting for 2.96–9.88% of the total variability. The three-way interaction among persons, sessions, and traces in addition to the residual came third, accounting for 2.12–2.89% in the three sitting postures.

### 3.2. Results of the D-Study

The results of the D-study showed excellent reliability coefficients of the SOL H_max_ measurements for the three sitting postures for two sessions and five measurement traces. G and Φ coefficients ranged from 0.943 to 0.980 and from 0.943 to 0.979, respectively. Table S1 shows the values of the reliability coefficients for the three sitting postures with alterations of the number of sessions and traces, which ranged from 0.892 to 0.988 for G coefficients and from 0.892 to 0.986 for Φ coefficients, indicating good to excellent reliability.

## 4. Discussion

Four main findings can be drawn from this study. First, among-subjects variance (i.e., true score variance) of the SOL H-reflex recordings accounted for the highest percentages of the total measurement variability. Second, the greatest amount of measurement error was caused by person-by-session interaction. Third, SOL H-reflex amplitude recordings in sitting positions showed good to excellent reliability. Fourth, recording five traces of the SOL H-reflex amplitude was sufficient to obtain good to excellent reliability during any of the three sitting postures.

The results of this study indicate that the largest portion of variability in the SOL H-reflex amplitude in each sitting position was due to the true score (among-subjects) variability (87.1 to 93.6%). These percentages are comparable with those reported by Handcock et al. [25] for the SOL H-reflex amplitude measurements obtained during standing position. In their study, they found that among-subject variance accounts for 98% of the total measurement variability.

The highest portion of measurement error in the SOL H-reflex amplitude recordings during the three sitting postures was derived from the interaction between persons and sessions. This source of error accounted for 2.96 to 9.88% of the total variability of measurements and was mainly evident in slouched sitting. This result is probably attributed to the minimal difference in the degrees of hip joint angle between the first and second sessions. In slouched sitting, participants had the opportunity to alter the hip joint angle to assume their most comfortable position. Therefore, the hip joint angle possibly had minimal change from session 1 to session 2. The degree of hip joint angle has been reported to significantly affect the excitability of the SOL H-reflex [5]. By contrast, in erect and slumped sitting postures, the hip joint was maintained at 90° during the two sessions. Therefore, standardizing the sitting posture when recording the SOL H-reflex amplitude would enhance the reliability of measurements when it is tested at separate sessions/occasions.

Between 2.12 and 2.89% of the total variability in the SOL H-reflex amplitude measurements was due to the three-way interaction among persons, sessions, and traces, in addition to other sources of variability that were not included in this analysis and/or random error. This source of variability was suggested to be the time of day in which the SOL H-reflex was recorded. Some individuals were tested in the morning, and others were tested later in the day at their convenience. This might cause some biological or cognitive state differences among the participants, which may lead to fluctuations in α-MN excitability [25,26]. Future studies may consider including the time of day as a potential source of variation when evaluating the reliability of SOL H-reflex amplitude recording.

Recording consistent SOL H-reflex amplitude traces is sometimes challenging during loaded conditions (e.g., standing) as compared with unloaded conditions (e.g., lying) due to some stabilizing and postural factors [27]. In this study, the trace-to-trace variability of the SOL H-reflex amplitude was minimal in the three sitting postures (≤0.18%), indicating the stability of trace recording during sitting position. This finding demonstrates minimal unnecessary fluctuations in the excitability of α-MNs caused by descending commands and peripheral inputs during sitting. Moreover, a minimal amount of measurement error of the SOL H-reflex amplitude was due to person by trace interaction (0.07–0.41%). This finding indicates the high within-session reliability of the SOL H-reflex amplitude recordings across subjects during the three sitting postures.

Electrical stimulation of the tibial nerve at the popliteal fossa could be uncomfortable for some individuals. For this reason, designing a reliable recording method of the SOL H-reflex with the least number of traces is important. The results of this study indicate that recording five traces is sufficient to obtain the reliable recording of the SOL H-reflex amplitude during any of the three sitting postures (G and Φ coefficients ranged from 0.943 to 0.980). Our results are in agreement with those in previous reports on the SOL H-reflex [13,14,25,28]. For example, Handcock et al. [25] reported a reliability coefficient of r = 0.96 when only four traces of the SOL H-reflex amplitude were recorded during standing position. Hopkins et al. [28] described the high reliability of the SOL H-reflex amplitude recordings obtained during standing and supine positions (intraclass correlation coefficients = 0.862 and 0.932, respectively) when as few as five traces are recorded. Merlet et al. [14] reported the correlation coefficient values of 0.57–0.91 for the SOL H-reflex amplitude recordings in sitting position with different knee angles when four traces were averaged. Similarly, Chen et al. [13] observed test–retest reliability coefficients between 0.62 and 0.97 for the SOL H-reflex amplitude measurements during sitting position with varied joint angles and isometric contractions at the ankle with four traces.

The results of the D-study revealed that adding more traces and sessions would yield slightly higher reliability of SOL H-reflex amplitude recordings in each sitting posture. For example, recording 10 measurement traces of the SOL H-reflex amplitude in comparison to five in one session would increase the G and Φ coefficients up to 0.3%. Similarly, performing three sessions of recordings would improve the reliability by 2.3–6.9% while recording only five measurement traces of the SOL H-reflex amplitude at each session. However, these changes in reliability associated with the increase in the number of sessions and traces are negligible given the fact that five traces of the SOL H-reflex amplitude would provide adequate reliability.

A possible limitation of this study is the gender of the participants. The participants were all males, which may limit the generalizability of the results. However, gender has been reported to have no significant effect on H-reflex studies [29]. Including both genders in future studies is recommended to confirm the applicability of the current findings in females. Another limitation is that we did not evaluate the reliability of the SOL H_max_/M_max_ ratio. Although it has been documented that both the H_max_ and H_max_/M_max_ ratio can be modulated by equal factors and convey similar information [10,30], we recommend evaluating the reliability of the H_max_/M_max_ ratio as well to confirm the findings of this study. Finally, the hip joint angle was not recorded during slouched sitting in the current study. Measuring the hip joint angle during slouched sitting may have provided useful information about the participants’ tendency toward finding the same hip angle from one recording session to another. Furthermore, such recordings might help in detecting any relationship between the angle and reliability of recordings.

## 5. Conclusions

This study suggests that recording five traces of the SOL H-reflex amplitude is sufficient to obtain high reliability during erect, slumped, and slouched sitting postures. While the stability of recordings was higher in erect and slumped sitting postures than in slouched sitting posture, the reliability in the three sitting postures is comparable. The greatest amount of measurement error in the three sitting positions is possibly caused by differences in the testing position from the first to the second session. Standardizing the sitting posture and joint position would yield the relatively higher reliability of the SOL H-reflex amplitude recordings, particularly when conducted on separate occasions.

## Figures and Tables

**Table 1:** Anthropometric data of the participants.

Variable	Mean	SD	Range
Age (year)	26.6	5.16	20–37
Height (cm)	173.8	5.5	163–180
Weight (kg)	70.2	9	47.1–82.1
BMI (kg/m^2^)	23.2	3	15.8–26.4

BMI, body mass index; SD, standard deviation.

**Table 2:** Sources of variability of Soleus (SOL) Hoffmann reflex (H-reflex) amplitude recordings identified and included in the analysis.

Source of Variability	Definition	Potential Source of Variability
P	- Person-to-person variability in SOL H-reflex amplitude, averaging over sessions and traces. - Due to true score variability.	Actual differences in the degree of α-MN excitability from one person to another.
S	- Session-to-session variability in SOL H-reflex amplitude, averaging over persons and traces. - Due to measurement error.	Differences in testing position from the first to the second session across all persons.
T	- Trace-to-trace variability in SOL H-reflex amplitude, averaging over persons and sessions. - Due to measurement error.	Sudden body movements during recordings occurred in all persons in both sessions.
PS	- SOL H-reflex amplitude variability in some persons from the first to the second session. - Due to measurement error.	Differences in testing position from the first to the second session in some persons.
PT	- SOL H-reflex amplitude traces variability in some persons within the same session. - Due to measurement error.	Sudden body movements during recordings occurred in some persons within the same session.
ST	- SOL H-reflex amplitude traces variability from the first to the second session across all persons. - Due to measurement error.	Sudden body movements during recordings from the first to the second session occurred in all persons.
PST, e	- SOL H-reflex amplitude traces variability from one session to another and from one person to another, plus the residual. - Due to measurement error.	Interaction among all sources of variability plus other sources not included in the analysis (e.g., time of day), and/or random error (e.g., interruption of the test by a phone call).

P, person; S, session; T, trace; e, residual.

**Table 3:** Means ± standard deviations of the SOL H-reflex amplitudes in millivolt for traces and sessions in each sitting posture.

	Erect		Slumped		Slouched	
	Session 1	Session 2	Session 1	Session 2	Session 1	Session 2
Trace 1	5.75 ± 2.71	5.60 ± 2.55	5.65 ± 2.33	5.56 ± 2.59	5.93 ± 2.25	6.00 ± 2.59
Trace 2	5.59 ± 2.54	5.66 ± 2.51	5.63 ± 2.53	5.40 ± 2.36	6.00 ± 2.51	6.00 ± 2.34
Trace 3	5.65 ± 2.69	5.33 ± 2.25	5.65 ± 2.60	5.30 ± 2.45	6.21 ± 2.28	5.99 ± 2.46
Trace 4	5.59 ± 2.71	5.39 ± 2.09	5.63 ± 2.39	5.15 ± 2.14	5.97 ± 2.41	5.99 ± 2.28
Trace 5	5.44 ± 2.75	5.18 ± 2.14	5.65 ± 2.34	5.36 ± 2.25	5.91 ± 2.40	6.02 ± 2.64

**Table 4:** Estimated variance components and percentages of variability for each source of variability in the three sitting postures.

Source of Variability	Erect		Slumped		Slouched	
	VC	%	VC	%	VC	%
P	5.801	92.39	5.425	93.63	5.104	87.17
S	0.000	0.00	0.021	0.37	0.000	0.00
T	0.011	0.18	0.000	0.00	0.000	0.00
PS	0.308	4.91	0.171	2.96	0.578	9.88
PT	0.026	0.41	0.023	0.40	0.003	0.07
ST	0.000	0.00	0.000	0.00	0.000	0.00
PST, e	0.133	2.12	0.153	2.64	0.169	2.89

VC, variance component; %, percentage of variability; P, person; S, session; T, trace; e, residual.

## Supplementary Materials

The following are available online at https://www.mdpi.com/2076-3271/8/4/48/s1, Table S1: G and Φ coefficients, standard errors of measurement, and 95% confidence intervals for the SOL H_max_ recordings in the three sitting postures with various levels of sessions and traces.

## References

[1] Burke D. (2016). Clinical uses of H reflexes of upper and lower limb muscles. Clin. Neurophysiol. Pract..

[2] Gajewski J., Mazur-Różycka J. (2016). The H-reflex as an Important Indicator in Kinesiology. Hum. Mov..

[3] Alrowayeh H.N., Sabbahi M.A., Etnyre B. (2005). Soleus and vastus medialis H-reflexes: Similarities and differences while standing or lying during varied knee flexion angles. J. Neurosci. Methods.

[4] Chen Y.S., Zhou S., Cartwright C. (2014). Effects of Ankle Joint Position and Submaximal Muscle Contraction Intensity on Soleus H-reflex Modulation in Young and Older Adults. Mot. Control..

[5] Knikou M., Rymer Z. (2002). Effects of changes in hip joint angle on H-reflex excitability in humans. Exp. Brain Res..

[6] Chen Y.-S., Zhou S., Cartwright C. (2015). Modulation of Soleus H-Reflex during Shortening and Lengthening Muscle Actions in Young and Older Adults. Chin. J. Physiol..

[7] Ken’ichi E., Yukio O., Yoshinori K., Tadaaki M., Satoshi I., Atsunori K., Daisaku M. (2003). Effect of weight bearing on the soleus H-reflex during upright standing under the head-out water immersion condition in humans. Environ. Med..

[8] Koceja D.M., Trimble M.H., Earles D.R. (1993). Inhibition of the soleus H-reflex in standing man. Brain Res..

[9] Schieppati M. (1987). The Hoffmann reflex: A means of assessing spinal reflex excitability and its descending control in man. Prog. Neurobiol..

[10] Ali A.A., Sabbahi M.A. (2000). H-reflex changes under spinal loading and unloading conditions in normal subjects. Clin. Neurophysiol..

[11] Miyoshi T., Nozaki D., Sekiguchi H., Kimura T., Sato T., Komeda T., Nakazawa K., Yano H. (2003). Somatosensory graviception inhibits soleus H-reflex during erect posture in humans as revealed by parabolic flight experiment. Exp. Brain Res..

[12] Niazi I.K., Türker K.S., Flavel S., Kinget M., Duehr J., Haavik H. (2015). Changes in H-reflex and V-waves following spinal manipulation. Exp. Brain Res..

[13] Chen Y.-S., Zhou S., Cartwright C.M., Crowley Z., Baglin R., Wang F. (2010). Test–retest reliability of the soleus H-reflex is affected by joint positions and muscle force levels. J. Electromyogr. Kinesiol..

[14] Merlet A.N., Cattagni T., Cornu C., Jubeau M. (2018). Effect of knee angle on neuromuscular assessment of plantar flexor muscles: A reliability study. PLoS ONE.

[15] Al-Jawayed I. (1999). The H-reflex modulation in lying and a semi-reclining (sitting) position. Clin. Neurophysiol..

[16] Claus A., Hides J.A., Moseley G.L., Hodges P.W. (2016). Thoracic and lumbar posture behaviour in sitting tasks and standing: Progressing the biomechanics from observations to measurements. Appl. Ergon..

[17] Hey H.W.D., Wong C.G., Lau E.T.-C., Tan K.-A., Lau L.-L., Liu K.-P.G., Wong H.-K. (2017). Differences in erect sitting and natural sitting spinal alignment—Insights into a new paradigm and implications in deformity correction. Spine J..

[18] Huang M., Hajizadeh K., Gibson I., Lee T. (2016). Analysis of compressive load on intervertebral joint in standing and sitting postures. Technol. Health Care.

[19] Wilke H.-J., Neef P., Caimi M., Hoogland T., Claes L.E. (1999). New In Vivo Measurements of Pressures in the Intervertebral Disc in Daily Life. Spine.

[20] Nalty T., Sabbahi M. (2001). Electrotherapy: Clinical Procedures Manual.

[21] Aymard C., Katz R., Lafitte C., Lo E., Pénicaud A., Pradat-Diehl P., Raoul S. (2000). Presynaptic inhibition and homosynaptic depression: A comparison between lower and upper limbs in normal human subjects and patients with hemiplegia. Brain.

[22] Brennan R.L. (2001). Generalizability Theory.

[23] Shavelson R.J., Webb N.M. (1991). Generalizability Theory: A primer.

[24] Koo T.K., Li M.Y. (2016). A Guideline of Selecting and Reporting Intraclass Correlation Coefficients for Reliability Research. J. Chiropr. Med..

[25] Handcock P., Williams L.R., Sullivan S.J. (2001). The reliability of H-reflex recordings in standing subjects. Electromyogr. Clin. Neurophysiol..

[26] Alrowayeh H.N., Sabbahi M.A. (2006). Vastus Medialis H-Reflex Reliability during Standing. J. Clin. Neurophysiol..

[27] Mynark R.G. (2005). Reliability of the soleus H-reflex from supine to standing in young and elderly. Clin. Neurophysiol..

[28] Hopkins J.T., Ingersoll C.D., Cordova M.L., Edwards J.E. (2000). Intrasession and intersession reliability of the soleus H-reflex in supine and standing positions. Electromyogr. Clin. Neurophysiol..

[29] Bodofsky E.B. (1999). Contraction-induced upper extremity H reflexes: Normative values. Arch. Phys. Med. Rehabil..

[30] Alrowayeh H.N., Sabbahi M.A., Etnyre B. (2011). Similarities and Differences of the Soleus and Gastrocnemius H-reflexes during Varied Body Postures, Foot Positions, and Muscle Function: Multifactor Designs for Repeated Measures. BMC Neurol..

