# Supplementary material for: Reliability of the Soleus H-Reflex in Different Sitting Postures
Source: Med Sci (Basel). 2020 Nov 25;8(4):48. doi: 10.3390/medsci8040048 (PMC7712283; doi:10.3390/medsci8040048)
Supplement: Supplementary file 1 [file medsci-08-00048-s001.pdf]

Table S1. G and Φ coefficients, standard errors of measurement, and 95% confidence intervals for the SOL H_{max} recordings in the three sitting postures with various levels of sessions and traces.

		**Erect**						**Slumped**						**Slouched**					
**S**	**T**	**G**	**SEM**	**CI**	**Φ**	**SEM**	**CI**	**G**	**SEM**	**CI**	**Φ**	**SEM**	**CI**	**G**	**SEM**	**CI**	**Φ**	**SEM**	**CI**
1	5	0.944	0.58	1.14	0.944	0.58	1.14	0.963	0.45	0.88	0.959	0.47	0.92	0.892	0.78	1.53	0.892	0.78	1.53
1	7	0.945	0.57	1.12	0.945	0.57	1.12	0.965	0.44	0.86	0.961	0.46	0.90	0.894	0.77	1.51	0.894	0.77	1.51
1	10	0.947	0.56	1.10	0.946	0.57	1.12	0.966	0.43	0.84	0.962	0.45	0.88	0.895	0.77	1.51	0.895	0.77	1.51
2	5	0.971	0.41	0.80	0.970	0.41	0.80	0.980	0.32	0.63	0.979	0.34	0.67	0.943	0.55	1.08	0.943	0.55	1.08
2	7	0.971	0.40	0.78	0.971	0.41	0.80	0.981	0.31	0.61	0.980	0.33	0.65	0.944	0.54	1.06	0.944	0.54	1.06
2	10	0.972	0.40	0.78	0.972	0.40	0.78	0.982	0.30	0.59	0.980	0.32	0.63	0.944	0.54	1.06	0.944	0.54	1.06
3	5	0.980	0.34	0.67	0.979	0.34	0.67	0.986	0.26	0.51	0.985	0.28	0.55	0.961	0.45	0.88	0.961	0.45	0.88
3	7	0.980	0.33	0.65	0.980	0.33	0.65	0.987	0.26	0.51	0.986	0.27	0.53	0.962	0.44	0.86	0.962	0.44	0.86
3	10	0.981	0.33	0.65	0.981	0.33	0.65	0.988	0.25	0.49	0.986	0.26	0.51	0.962	0.44	0.86	0.962	0.44	0.86

SEM, standard errors of measurement; CI, confidence intervals; S, sessions; T, traces.
